# Family-Centered Interventions and Quality of Life of Clients with Ostomy

**DOI:** 10.1155/2022/9426560

**Published:** 2022-08-29

**Authors:** Arash Golpazir-Sorkheh, Teimor Ghaderi, Saeed Mahmoudi, Khalil Moradi, Amir Jalali

**Affiliations:** ^1^Department of General Surgery, School of Medicine, Kermanshah University of Medical Sciences, Kermanshah, Iran; ^2^Department of Psychiatric Nursing, School of Nursing and Midwifery, Kermanshah University of Medical Sciences, Kermanshah, Iran; ^3^Substance Abuse Prevention Research Center, Research Institute for Health, Kermanshah University of Medical Sciences, Kermanshah, Iran; ^4^Department of Nursing, School of Nursing and Midwifery, Kermanshah University of Medical Sciences, Kermanshah, Iran

## Abstract

**Background:**

Family-centered intervention can be used as a therapeutic intervention to improve the quality of life (QOL) in clients with ostomy. This study aimed to determine the effects of family-centered intervention on the QOL in ostomy clients.

**Methods:**

A quasi-experimental study was carried out with participation of 70 clients with colostomy and 70 caregivers (family members). The participants were selected through convenient sampling and randomly allocated into the experimental and control groups. The experimental group received family-centered education. The education program included four sessions, 50–60 min each, that were implemented in two weeks at hospital wards or clients' houses for the clients' companions. Afterwards, the caregivers implemented the care at home for one month. The subjects in the control group received routine care before being discharged. The QOL of the clients in both groups was measured using the city of hope-QOL-ostomy questionnaire before and one month after the intervention.

**Results:**

The mean scores of the QOL after family-centered intervention in the experimental and control groups increased from 197.97 to 207.49 and from 195.2 to 199.03, respectively. The paired *t*-test showed a significant change in the experimental and control groups after the intervention at a confidence level of 95% (*p*=0.0001; *p*=0.002). In addition, after the intervention, however, there was a significant difference between the two groups in all these areas except for social aspects (*p*=0.007).

**Conclusion:**

Family-centered intervention can be used as a therapeutic intervention to improve the QOL in clients with ostomy. The intervention was effective in the physical, spiritual, psychological, and social health of these clients.

## 1. Introduction

An ostomy is a prosthetic medical device that creates a way of collecting wastes from the colon or bladder and depending on the organ that is affected; it is called colostomies or urostomies [1]. The surgery might be a measure to ensure the survival of the patient or to improve the QOL of the patient, and in either case, the patient faces immense mental pressure [2].

While the statistics of colorectal clients is easily accessible at the global scale, there is no international statistics report of ostomy [3]. More than one million in the United States [4] and 102000 individuals in the UK use colostomy [5]. These numbers are growing year by year so that 100000 in the United States [6] and 13500 in the UK start using colostomy every year [7]. There is no reliable statistics of ostomy clients in Iran; however, according to the latest report by the Iran Ostomy Society, there are about 30000 clients with ostomy in Iran [8].

Studies have shown that depression, loneliness, suicidal thoughts, low self-esteem, and avoiding social activities are common in these clients [9, 10]. In addition, these clients are usually worried about intestinal gas, diarrhea smell, ostomy leakage, and constant dissatisfaction of appearance and mental image of the body, which are of the common problems of these clients [11]. Anxiety and feeling shameful because of using ostomy create changes in the lifestyle of these individuals that appears in areas such as ability to find a job, reluctance to travel, and negative self-mental image. In addition, the patient's feeling about the physical changes might affect the way they treat their friends and family, which might lead to problems in social and marital lives [12].

All aspects of QOL in the clients who undergo ostomy surgery are affected [13]. In terms of physical aspect, urination and sexual functions are affected; in terms of psychological aspect, depression, loneliness, suicidal thoughts, humiliation, and low self-confidence are very common; and in terms of social aspects, a decrease in participation in social and leisure activities is notable [14]. In addition, these clients deal with skin side effects such as wounds and infection; feces smell, tympanites, and discharge of smelly gasses; nutritional problems; heavy medical costs; and changes in defection, dressing, exercising, and pregnancy [5]. Tsunoda et al. argued that ostomy attenuates the QOL of individuals so that clients who used to have a good QOL complain about the decline in the quality of their lives after using ostomy [15]. Yau et al. reported that ostomy surgery has a notable negative effect on the QOL of the patient [16].

According to the mentioned cases, it is vital to provide the required care to patients with ostomy [17]. Studies have shown that proper family care [18] and educating patients about self-care can improve the QOL notably [19]. Therefore, educating family members and the patients can have an impact on their QOL [17–19]. Using the results from different studies in this area, nurses can have a deeper knowledge of the challenges and the factors in the QOL of clients with ostomy. Through this, they can introduce better care educational programs for the clients and their families [20]. Family-centered empowerment model is designed in Iran for chronic clients, and it has been used for different diseases [3, 21]. The main objective of the family-centered empowerment model is to empower the family's (including patient and family members) QOL. This model is based on qualitative research based on a grounded theory approach including concept formation, concept development, definition of psychosocial processes, and inferring the central variable (family-centered empowerment). The model has been successfully implemented for the improvement of QOL in clients with different diseases [3, 21–25]. Family-centered care method empowers individuals and families and improves their independence. It supports family's participation in decision-making and providing care so that the family and patient's choices, values, beliefs, and cultural background are respected [26]. Nurses can employ these findings to have a deeper insight into the challenges and factors in the QOL of clients with ostomy. Through this, they can adopt and implement more effective care educational programs for clients and their families [27].

Marion concluded that family members of clients under intensive care felt being more useful when they were allowed to participate in providing care. In addition, the connection between the family and nurses created a participatory approach that made providing daily care to the patient more successful [25]. Therefore, more effective and empowered participation of family members in the care program can be effective in the improvement of health condition and welfare of these clients. This study is an attempt to determine the effects of family-centered intervention on the QOL in clients with ostomy.

## 2. Materials and Methods

### 2.1. Setting

A quasi-experimental study was carried out on clients with ostomy in Kermanshah-based public and state hospitals.

### 2.2. Participants and Selection

In this study, the main participants were the patients, whose QOL was examined. In addition, the patients' companions also received the necessary education to take good care of the patients. Therefore, both the companions and the patients participated in this study. The participants were selected through convenient sampling and then randomly (tossing a coin) allocated to the experimental and control groups. Inclusion criteria were caregiver living with patient, no mental/physical impairment, chronic physical and psychological clients, no narcotic drug dependence, and not participating in similar programs (client and caregiver). The required information was collected from patients' files and interviews with them. Exclusion criteria were leaving the study, missing more than one educational session, an incident that may affect the QOL, and development of psychological diseases throughout the study.

The minimum sample size was determined based on a mean comparison formula for one quantitative trait with two groups (confidence factor (1-*α*) = 95%, power of test (1-*β*) = 90%) following Xu et al. [28]. The minimum sample size for each group was obtained equal to 30, and taking into account probable leaves, 35 individuals were selected for each group (35 clients with ostomy and 35 caregivers for each group).

### 2.3. Method

**Figure 1:**
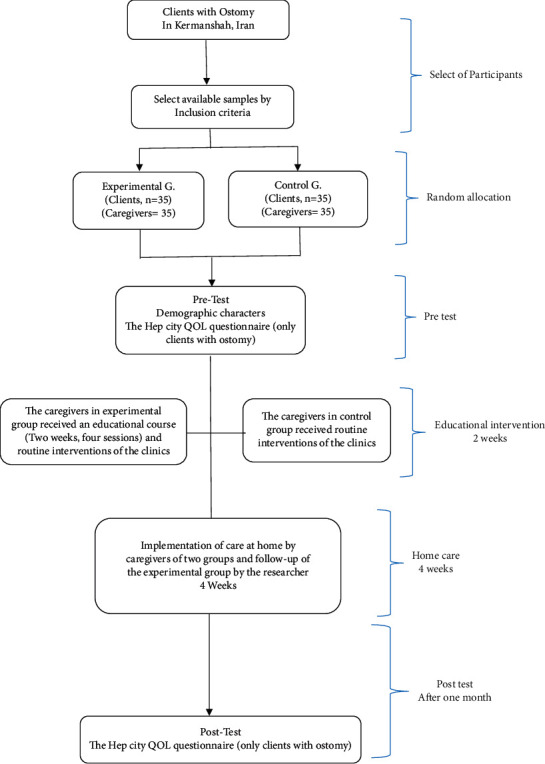
Study flowchart.

The participants signed a written letter of consent and then filled out a demographics form. QOL of the clients with ostomy was measured using the city of hope-QOL-ostomy questionnaire. The caregivers in the experimental group received an educational course designed based on nursing references and family-centered approach and family empowerment model for chronic clients [3]. The educational content was provided to experts (three surgeons, four faculty board members, and three operating room nurses) to examine qualitative content validity and face validity (using the opinions of 5 participants) of the tool, and their opinions were used to make the required modifications in the tool. The educational content included information about ostomy, side effects, nutrition, position change, infection prevention, intestine function control, activity, interaction with clients, and personal hygiene. The course was a four session's program (50–60 min each) that was implemented in two weeks either in the wards or in the houses for the caregivers. The educational content for the caregivers of the experimental group was the same, and each caregiver received four training sessions; however, the duration of the sessions varied between 50 and 60 minutes depending on the questions and the expected time needed by the caregivers. Afterwards, the participants were asked to implement the care program based on the plan and the researcher monitored the implementation of care using a checklist (previously confirmed by experts as a valid tool). In this study, all caregivers obtained the required grades and one to two extra training sessions had been made available in case some of the caregivers fail to obtain the minimum score. After making sure that the caregivers are empowered enough (checklist score >95%), they were asked to implement the care program for four weeks at home. An educational booklet was also provided to the participants along with a phone number to answer any question 24 h. Throughout this month, the researcher visited the participants at their houses four times to ensure continuity of the cares. In addition, while reviewing the care needed by the patients, questions and concerns of the patients and their companions were answered. We tried to prevent any contact between the caregivers and patients of the experimental and control groups during the intervention. Only the routine interventions of the clinics were provided for the caregivers and patients of the control group, which were also available for the patients and caregivers of the experimental group. One month after the completion of the family-centered intervention, the two groups (clients with ostomy) were again evaluated using the specific questionnaire of the QOL of Hep City. One month after completion of the family-centered care intervention, the two groups (clients with ostomy) filled out the city of hope-QOL-ostomy questionnaire. All stages of the intervention were done by a researcher (second author), and evaluations before and after the intervention were done by a senior nursing expert who was not part of the research team (Figure 1).

### 2.4. The City of Hope-Quality of Life-Ostomy Questionnaire

The data gathering tool was an ostomy clients' QOL scale designed by Hope City National Cancer Research Center, California, USA. The questionnaire contains 90 statements in three sections; section 1 (introduction) contains 13 questions on demographics and the disease. Section 2 (lifestyle impact) contains 34 multi-alternative questions on job, medical insurance, sexual activity, psychological concerns, dressing, diet, daily care for ostomy, and food groups. These questions are not scored and only give a description of the respondent. Section 3 (the effects of ostomy on the QOL) contains 43 questions on different aspects of physical health (1–11), psychological health (12–23), social health (25–36), and spiritual health (37–43) [29]. The questions in this part are scored based on Likert's rating scale of 0–10 and are used to calculate the mean score of the QOL. Some questions are scored inversely, so that a higher score indicates a better QOL in some questions and a lower QOL in some other questions. To calculate the score of the QOL, first by applying reverse changes to the questions with inverse scoring (questions 1–12, 15, 18, 19, 22–30, 32–34, and 37), the mean score of the QOL in each of its dimensions and also in general was determined. For the QOL as a whole and each of its dimensions, a minimum score (the worst) and the maximum score (the best) were zero and ten, respectively.

The validity of the tool has been determined based on face and content validity, and the reliability has been determined using test-retest and internal consistency. Cronbach's alpha of the tool is 0.95, and correlation coefficients for physical, psychological, social, and spiritual aspects of QOL have been reported equal to 0.82, 0.88, 0.83, and 0.78, respectively [20]. The tool has been validated for colostomy patient populations in Iran, and Cronbach's alpha for physical, psychological, social, and spiritual health aspects is 0.75, 0.85, 0.75, and 0.74, respectively [29]. In this study, the Cronbach's alpha coefficient for the tool was 0.874 and the subscales of physical, psychological, social, and spiritual health were 0.89, 0.691, 0.724, and 0.748, respectively.

### 2.5. Statistical Analysis

Data analysis was done using descriptive and analytical statistical methods in SPSS (v25). The Kolmogorov–Smirnov test was used to determine the normality of the data distribution. For data with normal distribution, the paired *t*-test was used to compare the desired quantitative trait before and after the intervention. The independent *t*-test was used to compare the mean of the desired quantitative trait in the experimental and control groups. Nonparametric tests, equivalent to Wilcoxon and Mann–Whitney, were used for the data without a normal distribution (*p* value = 0.05).

### 2.6. Ethical Consideration

After approval by the ethics committee (IR.KUMS.REC.1398.169), the objectives of the study were explained to the participants, they signed a letter of consent, the family members were asked for their permission before the researcher visited them at their home and implement family-centered intervention, the participants were ensured about the confidentiality of their information, and an ethical code was issued by the ethics committee of the university for the study. Given the fact that the intervention was effective and useful for the participants, the participants in the control group also took part in two educational sessions after the study.

## 3. Results

Totally, 70 clients and 70 caregivers took part in the study as the experimental and control groups (none of the participants left the study). Mean age of the clients was 51.86 ± 14.96 (min = 18; max = 92); mean age of the caregivers was 36.23 ± 11.2 (min = 18; max = 75); and mean body mass index (BMI) of the clients was 25.84 ± 3.27 (min = 21.26; max = 40.57). On average, the clients had used ostomy for 5.76 ± 2.03 days and the daily care time for ostomy was 118.21 ± 50.4 min. On average, the clients had 3.19 ± 2.18 children (min = 1; max = 9). For more demographic information, see Tables 1 and 2.

Based on the results of Kolmogorov–Smirnov (KS) tests and *p* value, normal distribution of the data of age of clients and caregivers, time duration of using ostomy, and BMI were supported. However, the time duration of daily care for the two groups and number of children in the experimental group were not normally distributed (*p* < 0.05). Based on the independent *t*-test, there was no significant difference between the experimental and control groups in terms of age of patient and caregiver, time duration of using ostomy, and BMI (*p* > 0.05). Therefore, the two groups were homogenous in terms of these variables. Based on the Mann–Whitney test, the two groups were homogenous in terms of number of children (*p*=0.451) and there was a significant difference between them in terms of daily time duration of care (*p*=0.043).

The KS test results showed the score of QOL, and the subscales had a normal distribution in the two groups before and after the intervention (*p* > 0.05). Only psychological scores in the control group and spiritual health in the experimental group were not normally distributed after the intervention (*p* < 0.05).

The mean scores of physical health, psychological health, and QOL of clients in the experimental and control groups were significantly different. However, the difference between the two groups as to social health and spiritual health was not significant. In addition, the two groups were not significantly different before the intervention in terms of physical, psychological, and spiritual health and QOL in general. However, after the intervention, the two groups were significantly different in terms of QOL and its subscales except for social health. The level of sig. for all the tests was 95% (Table 3).

The results of the mean scores of QOL before and after the intervention in the experimental group were (*M* = 195.2, SD = 14.49) and (*M* = 207.49, SD = 11.8), respectively, which imply significant differences that can be concluded to an improvement in QOL (*t* = −3.32, *p*=0.002). Also, the results of the mean scores of QOL before (*M* = 197.97, SD = 15.11) and after (*M* = 199.03, SD = 13.58) the intervention in the control group indicate significant differences that resulted in an improvement in QOL too (*t* = −5.3, *p*=0.0001). These differences in the mean scores between the two experimental and control groups were nonsignificant before (*t* = 0.78, *p*=0.44) and significant after (*t* = 2.87, *p*=0.007) the intervention (Table 3).

## 4. Discussion

As the results showed, the mean score of physical health in the clients of the experimental group had a significant increase compared with the control group. In addition, there was a significant increase in the mean score of physical health in the clients of the control group; however, this increase was less than that in the experimental group before and after the intervention. This increase in the clients of the control group can be explained by natural adaptation of the clients to ostomy. In addition, the control group received routine care and treatment during the study.

Naseh et al. found that clients with permanent ostomy did not have a good condition in terms of physical health and needed empowerment and care [20]. Rajabipour et al. reported that motivational interviews improved physical health of the clients with ostomy significantly [30]. Kalijzadeh Ganjalikhani et al. showed that structured education for ostomy care significantly improved physical health in clients with permanent ostomy [10]. Similar studies have shown that educating parents of children with ostomy can increase their care knowledge through family-centered empowerment to avoid ostomy side effects [23]. Clearly, there is good consistency between our results and other studies, which is an indicator of the right implementation of standard tools in this study. To explain the findings, we can say that the interventions were based on family-centered care for the clients and that family can play an effective role in improving client's condition [31].

Because of the family-centered intervention, the mean score of psychological health had a significant increase in the clients of the experimental group compared with the control group. The results supported a significant effect of family-centered intervention on psychological health of the clients. The increase in mean score in the clients of the control group can be the results of natural adaptation of the clients through time. Studies have emphasized on the effect of family-centered intervention on attenuating anxiety and stress [26] and improvement of self-confidence and self-efficacy [21] of clients. Here, family empowerment to provide proper therapeutic care and support to the clients, i.e., the key point in family-centered care program, was emphasized, which could be effective in the improvement of psychological health of the clients.

Nam et al. reported that clients with ostomy needed family and physician's support to adapt to the social and psychological challenges [32]. Hinton et al. found that family-centered care improved depression in clients [24]. To explain the findings, it is notable that along with empowering the family members to provide care to clients, family-centered care programs encompass several areas of psychological support. Additionally, studies have shown that family support has a significant relationship with the improvement of self-confidence [33], improvement of psychological condition [10, 32], and self-efficacy in patients [21].

The mean score of social health had a significant increase after family-centered intervention in the clients of the experimental group. This increase, however, was significantly higher than that in the control group, which might be due to the physical hardships and limitations in social interactions of the clients. Xu et al. concluded that self-efficacy interventions in clients with ostomy did not cause a significant change in the QOL in clients in the intervention group compared with the control group [28]. The results showed that the mean score of spiritual health had a significant increase after family-centered intervention. The results supported the effectiveness of family-centered intervention in the increase in spiritual health in care seekers. Several studies using other types of interventions have reported an improvement in different aspects of QOL along with attenuation of stress and anxiety in clients [28, 30, 32]; spirituality is one of these aspects.

As shown by the results, the scores of different aspects of QOL improved significantly after the intervention in the clients of the experimental group compared with the control group. The results supported the significant effect of family-centered intervention on the QOL in the clients. The increase in the mean scores in the control group might be due to the natural adoption process in the clients. Leyk et al. argued that time can be effective in gaining social support and improving health conditions of clients with ostomy and their families [34]. Koplin et al. maintained that psychological interventions can attenuate the decline in QOL in clients with ostomy [35]. To explain the findings, it is notable that educating family members and providing family-centered care can be effective in self-care skills and capabilities of clients with ostomy. As shown in [14], self-care activities can be effective in the improvement of QOL of clients with ostomy.

The mean scores in the clients of the control group increased during the four weeks of intervention. The natural adaptation process in the clients can explain this improvement so that they managed to handle many of their problems and improved their quality of lives. Time and education were effective in the QOL in clients with ostomy [36]. In addition, along with the client's attempt to solve their problem, social and family supports and routine educations and interventions can improve the QOL in clients [9, 35]. Several studies have shown that a variety of interventions are effective in the QOL in clients with ostomy [9, 14, 32, 35]. Our results also supported the effectiveness of family-centered intervention in the QOL of clients.

## 5. Limitation

A major limitation of the study was the large number of statements in the questionnaires that might have been tiring for the participants. To solve this, an assistant researcher was available to answer any question that the subjects could have. In addition, the questionnaires were filled out on different occasions with short time gaps. Another limitation was the challenge of finding clients with ostomy, which was done with the help of hospital officials. The study was carried out as an interventional study that needed a proper design and participation of the subjects. The study was part of a MSc. dissertation with a limited time. The sample groups were small, and the follow-up was limited to four weeks. Using a larger sample group and a longer follow-up term might lead to different and more reliable findings.

## 6. Conclusion

In general, family-centered interventions, as a therapeutic intervention, improved the QOL in clients with ostomy and improved their physical, social, spiritual, and psychological health. Therefore, the therapeutic intervention can be used by different surgery wards, nursing services, and social care services as an efficient intervention to improve QOL in clients.

## Figures and Tables

**Table 1:** Relative and absolute frequency of the experimental and control groups based on the demographics.

Variables		Control *N* (%)	Experimental *N* (%)	*X* ^2^/Fisher	*p*_value_
Gender	Male Female	17 (48.6) 18 (51.4)	20 (57.1) 15 (42.9)	0.516^*∗*^	0.18

Marital status	Unmarried Married	6 (17.1) 29 (82.9)	13 (37.1) 22 (62.9)	3.45^*∗∗*^	0.06

Educational status	Elementary level High school Higher education	25 (71.4) 6 (17.1) 4 (11.4)	28 (80) 4 (11.4) 3 (8.6)	0.713^*∗*^	0.7

Job	Employee Housewife Non-employed Self-employed	12 (34.3) 15 (42.9) 3 (8.6) 5 (14.3)	10 (28.6) 9 (25.7) 7 (20) 9 (25.7)	4.42^*∗*^	0.219

Residence	Urban Suburb Rural	14 (40) 12 (3.3) 9 (25.7)	11 (31.4) 17 (48.6) 7 (20)	1.47^*∗*^	0.48

Family income (monthly)	400$ 400-800$ 800-2000$	12 (34.3) 12 (34.3) 11 (31.4)	17 (48.6) 8 (22.9) 10 (28.6)	1.7^*∗*^	0.45

Ethnicity	Persian Azeri Kurd Lur	0 (0) 2 (5.7) 28 (80) 5 (14.3)	2 (5.7) 2 (5.7) 29 (82.9) 2 (5.7)		

Type of ostomy	Temporary colostomy Permanent colostomy Temporary ileostomy	17 (48.6) 4 (11.5) 14 (40)	20 (57.1) 7 (20) 8 (22.9)	4.48^*∗*^	0.214

Reason of ostomy	Cancer Inflammation disease Ileus Other	21 (60) 2 (5.7) 7 (20) 5 (14.3)	19 (54.3) 3 (8.6) 8 (22.9) 5 (14.3)	0.367^*∗*^	0.94

^*∗*^Exact chi-square test. ^*∗∗*^Exact Fisher test.

**Table 2:** Relative and absolute frequency of caregivers of the experimental and control groups based on the demographics.

Variables		Control *N* (%)	Experimental *N* (%)	*X* ^2^/Fisher	*P*_value_
Gender	Male Female	14 (40) 21 (60)	17 (48.6) 18 (51.4)	0.521^*∗*^	0.47

Marital status	Unmarried Married	6 (17.1) 29 (82.9)	13 (37.1) 22 (62.9)	3.54^*∗∗*^	0.06

Relation with clients	Parents child Sibling	0 (0) 32 (91.4) 3 (8.6)	3 (8.6) 27 (77.1) 5 (14.3)	3.9^*∗*^	

Job	Nonemployee Housewife Employed Self-employed	8 (22.9) 12 (34.3) 9 (25.7) 6 (17.1)	6 (17.1) 12 (34.3) 5 (14.3) 12 (34.3)	3.42^*∗*^	0.33

Educational status	Elementary level High school Higher education	12 (3.3) 13 (37.1) 10 (28.6)	14 (40) 13 (37.1) 8 (22.9)	0.37^*∗*^	0.83

^*∗*^Exact chi-square test. ^*∗∗*^Exact Fisher test.

**Table 3:** Comparison of mean scores of QOL and its subscales in clients of the experimental and control groups before and after intervention.

QOL_Aspect_	Group	Mean **±** SD		Paired *t*-test or Wilcoxon signed rank test	Sig.
		Pre	Post		
Physical health	Experimental	**9.69** **±** **38.11**	**14.05** **±** **47**	***T*** **=** **−3.22**	**0.003**
	Control	**10.43** **±** **39**	**8.14** **±** **40.86**	***T*** **=** **−2.93**	**0.006**
	Independent t-test	*T* = -0.37 Sig = 0.71	*T* = 2.24 Sig = 0.028		

Psychological health	Experimental	**5.6** **±** **60.29**	**5.09** **±** **64**	***T*** **=** **−11.125**	**0.0001**
	Control	**6.07** **±** **58.4**	**5.78** **±** **59.71**	***Z*** **=** **−3.27**	**0.01**
	Independent *t*-test/*U* Mann–Whitney	*T* = 1.35 Sig = 0.18	*Z* = -3.12 Sig = 0.002		

Social health	Experimental	**5.63** **±** **65.31**	**5.29** **±** **67.48**	***T*** **=** **−3.7**	**0.001**
	Control	**7.14** **±** **64.2**	**4.86** **±** **65.31**	***T*** **=** **0.365**	**0.717**
	Independent *t*-test	*T* = 0.72 Sig = 0.47	*T* = 1.79 Sig = 0.078		

Spiritual health	Experimental	**6.71** **±** **34.26**	**6.16** **±** **37.63**	***Z*** **=** **−4.56**	0.001
	Control	**5.93** **±** **33.6**	**5.74** **±** **33.37**	***T*** **=** **−0.173**	0.86
	Independent t-test/U Mann–Whitney	*T* = 0.434 Sig = 0.66	*Z* = -2.597 Sig = 0.009		

QOL	Experimental	**14.49** **±** **197.97**	**11.8** **±** **207.49**	***T*−/5.3**	**0.0001**
	Control	**15.11** **±** **195.2**	**13.58** **±** **199.03**	***T*** **=** **−3.32**	**0.002**
	Independent t-test	*T* = 0.78 Sig = 0.44	*T* = 2.87 Sig = 0.007		

## Data Availability

The datasets used and analyzed during this study are available from the corresponding author on reasonable request.

## Abbreviation

BMI: Body mass index
WHO: World Health Organization
QOL: Quality of life
KUMS: Kermanshah University of Medical Sciences.
